# Unexpected phenotype in a frameshift mutation of *PTCH1*

**DOI:** 10.1002/mgg3.987

**Published:** 2019-10-02

**Authors:** Benedetta Beltrami, Elisabetta Prada, Gianluca Tolva, Giulietta Scuvera, Rosamaria Silipigni, Daniela Graziani, Gaetano Bulfamante, Cristina Gervasini, Paola Marchisio, Donatella Milani

**Affiliations:** ^1^ Pediatric Highly Intensive Care Unit Department of Pathophysiology and Transplantation Università degli Studi di Milano Fondazione IRCCS Ca' Granda Ospedale Maggiore Policlinico Milano Italy; ^2^ Laboratory of Medical Genetics Fondazione IRCCS Ca' Granda Ospedale Maggiore Policlinico Milano Italy; ^3^ Department of Human Pathology Cytogenetic and Molecular Pathology ASST Santi Paolo e Carlo Milan Italy; ^4^ Department of Health Sciences Medical Genetics Università degli Studi di Milano Milano Italy

**Keywords:** 9q22.3 microdeletion syndrome, craniosynostosis, Gorlin syndrome, PTCH1

## Abstract

**Background:**

Gorlin syndrome, also known as basal cell nevus syndrome (BCNS), is a rare autosomal dominant genetic condition, characterized by the presence of multiple basal cell carcinomas at a young age, odontogenic keratocysts, skeletal anomalies, macrocephaly, and dysmorphisms. BCNS is mainly caused by mutations in *PTCH1*, an onco‐suppressor gene that maps at 9q22.3 region. A disease related to BCNS is the 9q22.3 microdeletion syndrome. This condition has an overlapping clinical phenotype with the BCNS, but it can present in addition: metopic craniosynostosis, overgrowth, obstructive hydrocephalus, developmental delay, intellectual disability, and seizures. This syndrome is caused by the deletion of a genomic region containing the *PTCH1* and the *FANCC*.

**Methods and Results:**

We report the case of an 11‐year‐old girl that came to our attention for overgrowth, dysmorphic features of the face, and craniosynostosis, but with a normal intellectual and motor development. At first we performed an array‐comparative genomic hybridization (aCGH) analysis. The analysis showed no copy number changes. Then, we performed the analysis of the *PTCH1* by next‐generation sequencing. This analysis showed a heterozygous frameshift mutation.

**Conclusion:**

This is the first case with a *PTCH1* point mutation with a 9q22.3 microdeletion syndrome phenotype. This finding may strengthen the importance of the role of the *PTCH1*, especially regarding the metopic craniosynostosis.

## Graphical abstract

We report the case of an 11‐year‐old girl that came to our attention for overgrowth, dysmorphic features of the face and craniosynostosis, but with a normal intellectual and motor development. This is the first case with a PTCH1 point mutation with a 9q22.3 microdeletion syndrome phenotype.

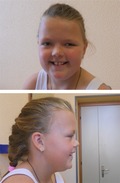

## 1 INTRODUCTION

Gorlin syndrome is a genetic multisystemic disorder; it is also called Basal Cell Nevus Syndrome (BCNS, MIM **#**109400). It has a prevalence of 1/50.000, both sexes are equally affected. The syndrome has an autosomal dominant inheritance, with a high penetrance and a variable expressivity (Reinders et al., 2018; Witmanowski, Szychta, Btochowiak, Jundzitt, & Czajkowski, 2017). The primary cause of the disease is a heterozygous mutation in the onco‐suppressor gene *PTCH1* (*protein patched homolog* 1, MIM* 601309). Many types of mutations can lead to the syndrome, including frameshift, missense, nonsense, and splicing mutations. Other genes that can cause the disease are *SUFU* (MIM* 607035) and less commonly *PTCH2* (MIM* 603673). Those genes are involved in the sonic hedgehog pathway. In the 30% of the patients, there is no family history while the 70% has at least one first‐degree relative with the BCNS (Evans et al., 2017). The syndrome owes its name to the presence of multiple basal cell carcinomas. The main age of onset of those tumors is 25 years of age, the number of them can be variable, from a few to thousands. Many systems can be involved, such as the skeletal (bifid, fused or missing ribs, congenital vertebral anomalies, kyphoscoliosis), the stomatologic system (keratocystic odontogenic tumors, dental ectopy, and agenesis), the central nervous system (calcification of the falx cerebri, congenital hydrocephalus, bridging of the sella turcica), and the ocular (cataract, coloboma, microphthalmia, strabismus). The phenotype of the patients with the syndrome is peculiar: they present hypertelorism, broad nasal bridge, frontal bossing, palmar/plantar pits, and macrocephaly. Furthermore, the patients affected by BCNS have an incremented risk to develop various types of tumors (medulloblastoma, ovarian, and cardiac fibromas; Evans et al., 2017; Şereflican et al., 2017; Witmanowski et al., 2017). The 9q22.3 microdeletion syndrome is a disease related to BCNS. It is a contiguous gene deletion syndrome, and the main genes involved in the condition are *PTCH1* and *FANCC* (MIM* 613899). The clinical manifestations are similar to those of BCNS; additional features that can be found in the syndrome, and not in BCNS are: metopic craniosynostosis, obstructive hydrocephalus, macrosomia, and intellectual disability (Muller et al., 2012; Reichert et al., 2015).

## 2 CLINICAL REPORT

**Figure 1:**
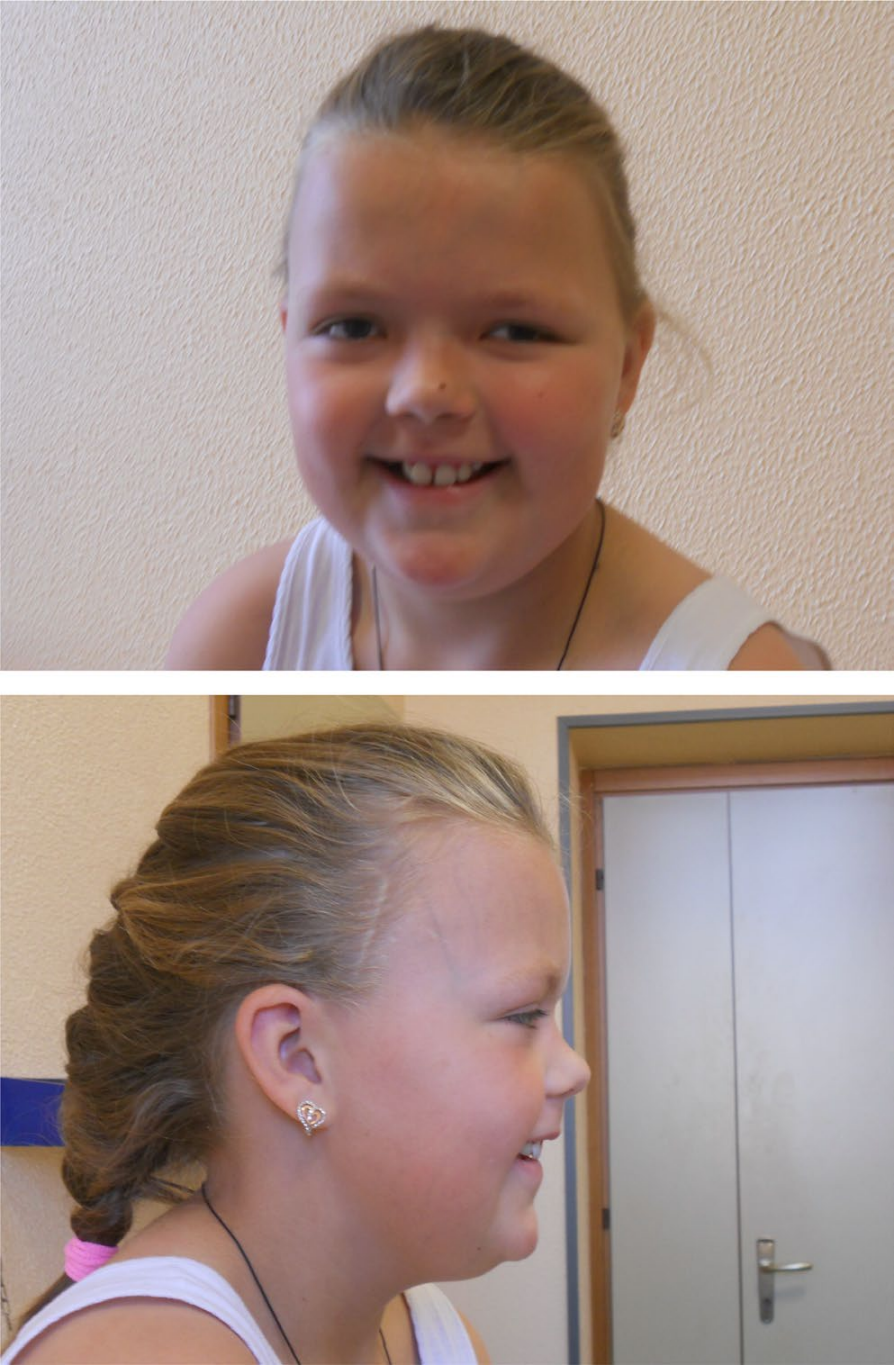
Photographs of the patient when she was 11 years old. Note, macrocephaly, big forehead, hypertelorism, saddle nose, and small mouth

Our case is an 11‐year‐old girl, with a silent family history and non‐consanguineous parents. Her pregnancy was normal, she was born at 41 weeks of gestational age by a C‐section. Her birth weight was 4.880 kg (>97°), her height was 57 cm (>97°), and her parents reported that the occipital‐frontal circumference was also >97°. At the age of 1 month, she was diagnosed with the craniosynostosis of all sutures, she underwent surgery at the age of 7 months. At 2 years of age, she was diagnosed with right vesicoureteral reflux, while the renal function was normal. When she was 6 years old, she started to present several jaw keratocysts, confirmed by histological examination. An orthopedic evaluation revealed a calcaneo‐navicular synostosis and a spinal X‐ray showed a vertebral schisis of the posterior arches of C7 and T1 and a transverse mega‐apophysis of C7. We evaluated the girl when she was 9 years old. Physical examination showed macrocephaly, a wide forehead, hypertelorism, saddle nose, and small mouth (Figure 1). Her weight was 54 kg (>97° centile), her height was 153.5 cm (>97° centile), and her occipital‐frontal circumference was 57.8 cm (>97°centile).

## 3 RESULTS OF GENETIC TESTING

The first diagnostic hypothesis was the 9q22.3 microdeletion syndrome, due to the presence of craniosynostosis and overgrowth associated with jaw keratocysts. The presence of craniosynostosis had already been investigated through the analysis of the *FGFR3* and the *FGFR2*, the result was normal. In order to confirm this hypothesis, an array‐comparative genomic hybridization (aCGH) was performed using a 180‐mer oligonucleotide probes technology (SurePrint G3 Human CGH 4x180K, Agilent Technologies) according to the manufacture's instruction. Raw data were generated using Agilent Feature Extraction and analyzed by Cytogenomics 3.0.4.1 using ADM‐2 algorithm (Agilent Technologies). To improve the accuracy of the results, the Diploid Peak Centralization algorithm was applied. The aberration filter was set to detect a minimum number of five consecutive probes/region and the minimum absolute average log ratio (MAALR) was ±0,25. A second analysis was run with a MAALR of ±0,15 and with a minimum number of five probes/region to detect low level mosaicism. Copy number variations were not reported if they coincided with the published DNA variants listed in the Database of Genomic Variants (http://projects.tcag.ca/variation/). Genomic coordinates are according to the 37 build (March 2009) of the Human Genome Reference consortium (GRch37/hg19). Unexpectedly, the result of the analysis was normal. The other diagnostic hypothesis was Gorlin syndrome. This is the reason why the analysis of the *PTCH1* by next‐generation sequencing was performed. This analysis showed a c.1502A duplication in exon 6. This variation causes a frameshift mutation p.(Val502Glyfs*13), that leads to the formation of a premature stop codon. This mutation has never been reported in literature, but mutations in the same region have been described as pathogenetic variants. We could not perform the segregation study because the parents denied the consent.

## 4 DISCUSSION

In literature, many cases of *PTCH1* mutations are described, and they have always been related to Gorlin syndrome. *PTCH1* is the first gene that clinicians analyze when the phenotype of the patient is consistent with BCNS: macrocephaly, jaw keratocysts, multiple basal carcinomas, and so on (Evans et al., 2017; Witmanowski et al., 2017). The syndrome has never been associated with craniosynostosis and overgrowth. This is the reason why our first diagnostic hypothesis was the 9q22.3 microdeletion syndrome. Those characteristics are indeed very well described in the 9q22.3 microdeletion syndrome (Muller et al., 2012; Reichert et al., 2015). This is the first case of a patient with a *PTCH1* point mutation and a 9q22.3 microdeletion syndrome phenotype (Table 1). At protein level, we hypothesized that the identified mutation can lead to the formation of a truncated protein without several domains. In particular, the predicted effect of mutation at protein level (p.(Val502Glyfs*13)) suggests that the sterol‐sensing domain (426‐616aa) is lacking. Functional studies of the variation show an alteration of the splicing. Those findings lead us to think that this mutation is pathogenetic. Conversely, the reported point mutations of *PTCH1*, associated with Gorlin phenotype, are in general localized in the second half of the gene, preserving the sterol‐sensing domain. This domain is important to sterol regulatory element‐binding protein (SREBP) cleavage activation. We can hypothesize that this domain can influence the correct cranial development and that the formation of the protein without this domain or the mRNA decay leading to a complete haploinsufficiency might favor additional phenotypic features such as the craniosynostosis, reported in the 9q22.3 deletion syndrome. Further studies are needed to prove this connection. Anyway, this finding may strengthen the importance of the role of the *PTCH1*, especially regarding the syndromic craniosynostosis.

**Table 1:** Summary of the features of Gorlin syndrome and 9q22.3 deletion syndrome

	Gorlin syndrome	9q22.3 deletion	Our patient
Multiple basal cell carcinomas	+	+	−
Odontogenic keratocyst	+	+	+
Skeletal anomalies	+	+	+
Palmoplantar pits	+	+	−
Calcification of the falx cerebri	+	+	−
Macrocephaly	+	+	+
Frontal bossing	+	+	+
Macrosomia	−	+	+
Metopic craniosynostosis	−	+	+
Obstructive hydrocephalus	−	+	−
Intellectual disability	−	+	−

## CONFLICT OF INTEREST

None declared.

## CONSENT

Written informed consent was obtained from the patient's parents for the publication of this report and any accompanying images.
